# The Hyperintense study: Assessing the effects of induced blood pressure increase and decrease on MRI markers of cerebral small vessel disease: Study rationale and protocol

**DOI:** 10.1177/23969873221100331

**Published:** 2022-05-12

**Authors:** Esther Janssen, Annemieke ter Telgte, Esmée Verburgt, Joost JA de Jong, José P Marques, Roy PC Kessels, Walter H Backes, Marnix C Maas, Frederick JA Meijer, Jaap Deinum, Niels P Riksen, Anil M Tuladhar, Frank-Erik de Leeuw

**Affiliations:** 1 Department of Neurology, Donders Institute for Brain, Cognition and Behaviour, Radboud University Nijmegen Medical Centre, Nijmegen, The Netherlands; 2 Donders Institute for Brain, Cognition and Behaviour, Radboud University Nijmegen, Nijmegen, The Netherlands; 3 VASCage GmbH, Research Centre on Vascular Ageing and Stroke, Innsbruck, Austria; 4 School for Mental Health & Neuroscience, Maastricht University Medical Center, Maastricht, The Netherlands; 5 Vincent van Gogh Institute for Psychiatry, Venray, The Netherlands; 6 Department of Medical Psychology and Radboudumc Alzheimer Center, Radboud University Medical Center, Nijmegen, The Netherlands; 7 Department of Medical Imaging, Radboud University Medical Center, Nijmegen, The Netherlands; 8 Department of Internal Medicine and Radboud Institute for Molecular Life Sciences, Radboud University Medical Center, Nijmegen, The Netherlands

**Keywords:** Small vessel disease, hypertension, blood brain barrier, DCE-MRI, cerebral blood flow, diffusion tensor imaging

## Abstract

**Background:**

Neuroimaging markers of cerebral small vessel disease (SVD) are common in older individuals, but the pathophysiological mechanisms causing these lesions remain poorly understood. Although hypertension is a major risk factor for SVD, the direct causal effects of increased blood pressure are unknown. The Hyperintense study is designed to examine cerebrovascular and structural abnormalities, possibly preceding SVD, in young adults with hypertension. These patients undergo a diagnostic work-up that requires patients to temporarily discontinue their antihypertensive agents, often leading to an increase in blood pressure followed by a decrease once effective medication is restarted. This allows examination of the effects of blood pressure increase and decrease on the cerebral small vessels.

**Methods:**

Hyperintense is a prospective observational cohort study in 50 hypertensive adults (18–55 years) who will temporarily discontinue antihypertensive medication for diagnostic purposes. MRI and clinical data is collected at four timepoints: before medication withdrawal (baseline), once antihypertensives are largely or completely withdrawn (*T* = 1), when patients have restarted medication (*T* = 2) and reached target blood pressure and 1 year later (*T* = 3). The 3T MRI protocol includes conventional structural sequences and advanced techniques to assess various aspects of microvascular integrity, including blood-brain barrier function using Dynamic Contrast Enhanced MRI, white matter integrity, and microperfusion. Clinical assessments include motor and cognitive examinations and blood sampling.

**Discussion:**

The Hyperintense study will improve the understanding of the pathophysiological mechanisms following hypertension that may cause SVD. This knowledge can ultimately help to identify new targets for treatment of SVD, aimed at prevention or limiting disease progression.

## Background

Cerebral small vessel disease (SVD) is an umbrella term covering a variety of pathologies that affect the small arteries, arterioles, and capillaries in the brain.^1^ SVD can be clinically covert, but is associated with cognitive decline, dementia, and disturbances in gait and mood.^1^ Moreover, most hemorrhagic strokes and a fourth of all ischemic strokes are caused by SVD.^1^

Small vessel pathology cannot be visualized in vivo with standard 1.5 or 3T Magnetic Resonance Imaging (MRI). SVD is therefore usually defined by tissue alterations on MRI thought to be a consequence of small vessel pathology, such as white matter hyperintensities (WMH), lacunes, and microbleeds.^2^ However, these MRI markers are likely the result of long ongoing pathological processes and studying them provides limited insights into their (early) pathogenesis. Endothelial dysfunction is hypothesized to play a key role in the pathophysiology of SVD and may explain the various pathologies seen in SVD patients, including loss of integrity of the Blood Brain Barrier (BBB), vessel wall stiffening, impairments in vasodilation, reduced cerebral blood flow, and increased inflammation.^2^ The relation between these pathological processes, the order in which they occur and the role they play in the etiology of SVD remains largely unknown.

To advance our understanding of SVD etiology and progression, research in young- and middle-aged adults at risk or with early-stage SVD is crucial. Hypertension is the most established risk factor for SVD, but evidence is based on prospective and cross-sectional studies.^3^ Higher blood pressure in midlife is shown to lead to higher SVD burden and smaller brain volumes at later age.^4^ Moreover, the presence of cardiovascular risk factors, including hypertension, at a young age (18–40 years), is associated with changes in vessel morphology (i.e. vessel density and vessel caliber) and higher WMH volume.^5^ This suggests that small vessel changes precede end-stage MRI markers and the accompanying clinical symptoms for decades, but the underlying mechanisms are unknown. In addition, the causal relation with hypertension is predominantly based on prospective cohort studies and trials that have shown slower SVD progression among those with lower blood pressure or active treatment with anti-hypertensives, but no studies have examined the actual effect of *increasing* blood pressure on MRI markers of SVD.

In this paper we describe the protocol of the Hyperintense study. We will apply an advanced MRI protocol to examine early functional and (micro)structural changes in young-and middle-aged adults with hypertension, the most important cardiovascular risk factor of SVD. Specifically, we will examine the effects of blood pressure increases in patients with hypertension who undergo a routine diagnostic work up which includes temporary withdrawal of their antihypertensives, followed by subsequent decrease after reinstatement of therapy. The design of this study, that is, temporal withdrawal of antihypertensive drugs in patients with refractory hypertension, allows assessment of the pathological mechanisms following blood pressure increase. At four time-points, advanced MRI sequences will be applied to probe potential changes in BBB integrity, microvascular perfusion, microstructural integrity, and functional connectivity.

## Methods

### Study population and design

Patients with hypertension (*n* = 50) will be recruited at the outpatient clinic of the Department of Internal Medicine of the Radboud University Medical Center (Radboudumc), which is a national referral center for patients with complex hypertension. Approximately 120 patients aged 18–55 years with hypertension are referred to the Radboudumc annually. These patients are often referred by general practitioners or other hospitals when blood pressure is not well-controlled (>140/90 mmHg) despite the use of three or more antihypertensive drugs or when there is clinical suspicion for secondary forms of hypertension. To determine if high blood pressure is caused by an overproduction of aldosterone in the adrenal gland (i.e. primary hyperaldosteronism), the plasma aldosterone/renin ratio (ARR) can be determined. Because many common hypertensive drugs interfere with this ratio, patients often have to discontinue antihypertensive drugs prior to screening or switch to drugs that are known not to affect ARR (i.e. doxazosin, verapamil, diltiazem, hydralazine) according to local protocols.^6^ Medication has to be stopped for at least 4 weeks (for mineralocorticoid receptor antagonists) or 2 weeks (for diuretics, Angiotensin Converting Enzyme (ACE) inhibitors, Angiotensin Receptor Blockers (ARBs)). This often leads to a temporary increase in blood pressure. After diagnostics are completed, medication is adjusted accordingly, and blood pressure levels drop again. This diagnostic protocol with temporary withdrawal of antihypertensive medication has been proven to be safe.^7^

Antihypertensive medication is discontinued in approximately 50% of patients referred to Radboudumc. We expect that at least 30% of these patients will be willing to participate in our study, making it feasible to include 50 patients within the planned 2.5 years. All participants will be asked to provide written informed consent. This study has been approved by the medical ethics committee region Arnhem-Nijmegen.

### Inclusion criteria

1. Age of 18–55 years
2. Antihypertensive drug treatment for hypertension
3. Temporary antihypertensive medication withdrawal as part of clinical diagnostic routine

### Exclusion criteria

Subjects will be excluded if they meet any of the following criteria:

1. A history of an ischemic or hemorrhagic stroke or transient ischemic attack (TIA). This is based on self-report and subsequent verification of available medical documentation.
2. Conditions leading to similar appearance of MRI markers as SVD, including:
  a. Large-artery disease, defined as stenosis >50% in the internal carotid artery or vertebral artery based on ultrasound collected at baseline and medical records
  b. Cardioembolism, defined as atrial fibrillation or other high risk cardioembolic conditions (based on medical history)
  c. Vasculitis
3. Major neurological/psychiatric diseases or other diseases that prevent long-term follow-up
4. Contraindications for 3T MRI
  a. Renal insufficiency (eGFR <30 ml/min, insufficient kidney function to receive gadolinium-based contrast agent for DCE-MRI)
  b. Pregnancy
5. Inability to give informed consent.

### Study visits

**Figure 1.**
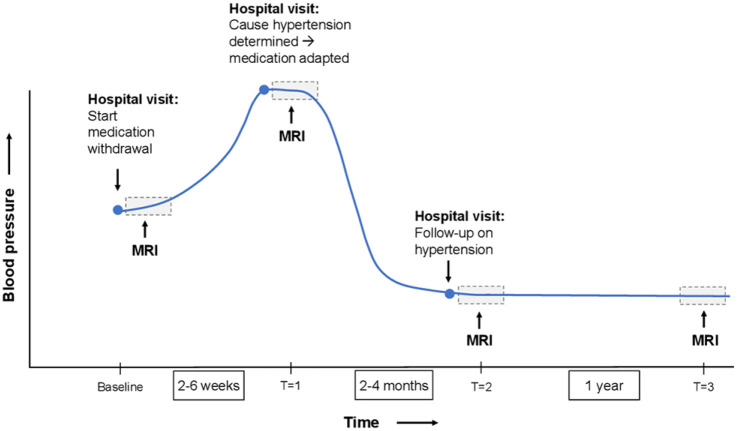
Schematic presentation of the design of the Hyperintense study.

Participants will be requested to complete four study visits, combined with routine visits to the outpatient clinic when possible (Figure 1). The baseline measurement is conducted just before antihypertensive medication is withdrawn (*T* = 0). The second study visit (*T* = 1) will take place approximately 2–6 weeks after antihypertensive medication withdrawal when blood pressure is increased. *T* = 2 is scheduled within 2–4 months once patients have reached their target blood pressure and blood pressure is stable. The final study visit takes place approximately 1 year after *T* = 2 (*T* = 3). After the final study visit, long-term future incident clinical events will be monitored by contacting the GP or treating physician of the participants. Table 1 shows the data that will be collected at each study visit.

**Table 1.** Schedule of all assessments in the Hyperintense study.

	Pre-visit	Baseline	*T* = 1	*T* = 2	*T* = 3
Screening					
Ultrasound of carotid arteries	X				
MRI		X	X	X	X
Cognitive assessment					
Full cognitive assessment		X		X	X
Test of attentional performance		X	X	X	X
Motor assessment					
Timed Up & Go test		X	X	X	X
Six-meter walk test		X	X	X	X
Physical assessment					
Blood pressure		X	X	X	X
Weight, length, BMI		X			X
Questionnaires					
Educational level		X			
Medical history		X			
Medication use		X	X	X	X
Substance use		X	X	X	X
Lifestyle and behavior		X	X	X	X
Blood sampling			X	X	

### Imaging protocol

During each study visit, a brain MRI scan will be performed on a 3T MRI system (MAGNETOM PrismaFit, Siemens Healthcare, Erlangen, Germany) using a 20 channel head-neck coil. The total imaging protocol will take approximately 75 min and includes the following sequences: 3D *T*1-weighted MP2RAGE, Fluid Attenuated Inversion Recovery (FLAIR), susceptibility-weighted imaging (SWI), multi-shell Diffusion Weighted Imaging (DWI), resting-state functional MRI (rs-fMRI), Intravoxel Incoherent Motion (IVIM), and Dynamic Contrast Enhanced (DCE) MRI. In addition, a B0 field map and a diffusion-weighted sequence with reversed phase-encoding direction were acquired for distortion correction of DWI, IVIM, and rs-fMRI scans. Acquisition parameters for all sequences are shown in Supplemental Table 1. DCE-MRI consists of successive slow, fast, and slow *T*1-weighted saturation recovery spoiled GRE pulse sequences.^8^ Gadobutrol contrast agent (0.1 mmol/kg) will be injected at a 3 ml/s rate followed by a 20 ml saline flush during the fast sequence. This macrocyclic agent is shown to be more stable than other gadolinium-base contrast agents and can therefore safely be used for multiple MRI assessments.^9^

### MRI processing and analysis

#### Structural MRI measures

##### MRI markers of SVD

Conventional MRI markers of SVD, such as WMH, lacunes, microbleeds, enlarged perivascular spaces, and recent infarcts will be examined following the STRIVE guidelines.^10^ WMH volume will be calculated using an in-house developed and validated technique that has previously been described.^11^

##### DWI

Diffusion-weighted images will be processed according to a previously described protocol.^12^ In short, images will be visually inspected to exclude major artifacts. The following preprocessing steps will be applied: denoising, removal of Gibbs artifacts and correction for head motion, susceptibility-induced distortions, and eddy current-induced distortions. To do this we will use the Functional Magnetic Resonance Imaging of the Brain (FMRIB) software library (FSL; v5.0, topup, eddy) and MRtrix3 (mrtrix.org/, dwidenoise, mrdegibbs).^13,14^ BET (FSL) is used to extract brain tissue, after which the diffusion tensor metrics (FA and MD) will be calculated using DTIFIT (FSL).

Next to these common DTI-derived measures, the Peak Width of Skeletonized Mean Diffusivity (PSMD) will be calculated. PSMD is based on skeletonization and histogram analysis and is calculated as the difference between the 95th and 5th percentiles of MD values within the masked skeleton.^15^ This approach is suggested to improve detection of subtle diseases in the brain.^15,16^ PSMD will be calculated using a fully automated pipeline that was previously described.^15^

#### Functional MRI measures

##### IVIM

IVIM is a DWI-technique that is used to study both microvascular perfusion and changes in microstructural tissue properties, which is especially useful in SVD research since both are impaired in this disease.^17^ Processing of IVIM images has been previously described.^17^ In short, preprocessing steps will include visual inspection of image quality, distortion corrections and head motion correction. To model the diffusion-attenuated signal, a two-compartment diffusion model is used that describes vascular and nonvascular compartments.^18^ Fitting of the model will be performed on a voxel-by-voxel basis using a previously described two-step method.^19^ Measures derived from this model include: the perfusion volume fraction *f*, the diffusion coefficient of parenchymal water *D*, and the pseudodiffusion coefficient of circulating blood D*. Furthermore, fD* is a blood perfusion related measure that will be calculated.^20^

##### DCE-MRI

DCE-MRI images will be processed according to previously described protocol by performing pharmacokinetic modeling and histogram analyses.^21^ Images are segmented into white and gray matter using the *T*1-weighted image and WMH are segmented on FLAIR images. After coregistration of *T*1-weighted images and FLAIR, Normal Appearing White Matter (NAWM), WMH, cortical gray matter, and deep gray matter are selected as regions of interest (ROI).

To calculate the concentration of the contrast agent in tissue, the relative signal enhancement and *T*1 maps derived from the MP2RAGE sequence will be used.^22^ The vascular input function used to calculate the contrast concentration in blood plasma will be derived from the superior sagittal sinus.^23^ The slope and intercept of the graphical Patlak model will be used to calculate the leakage rate (transfer constant K_i_).^24^ A histogram is created for the K_i_ values in each ROI in a voxel-wise manner. Two measures to quantify BBB leakage are derived from these histograms: the mean transfer constant K_i_ as a measure of leakage rate and the area under the histogram curve as a measure of tissue volume of leaking microvessels (V_l_). These measures will be calculated for all ROIs. *T*1 values in these ROIs will be computed at the individual level to evaluate the interaction between hypertension and *T*1, a marker of water mobility.

##### Resting-state fMRI

Processing of resting-state functional MRI (rs-fMRI) includes the following steps: removal of artifacts, correction for slice time and head motion, co-registration of functional and structural images, normalization of subject brain to Montreal Neurological Institute (MNI) space and spatial filtering. Graph theory will be used to examine functional brain networks using a previously described protocol.^25^ In short, 264 functional areas will be used as network nodes in the cerebral cortex, subcortical structures, and cerebellum.^26^ We will define five functional networks: a global brain network, the default mode network, the fronto-parietal task control network, the somatosensory-motor network of the hand, and the visual network.^25^ For these networks, we will calculate the weighted global efficiency and the weighted clustering coefficient, since these measures have previously been shown to be sensitive to structural network abnormalities in SVD patients.^27^

### Primary outcomes

Primary outcomes of this study will be the changes in MRI outcomes after withdrawal of antihypertensive medication (highest blood pressure) and subsequent restart (lowest blood pressure). MRI outcomes include WMH volume, PSMD, BBB leakage rate and volume, IVIM outcomes (D, fD*), PSMD, network global efficiency, and clustering coefficients and are described in more detail in the “Imaging Protocol” section.

Secondary study outcomes include:

‒ Effects of antihypertensive medication withdrawal and restart on cognitive and motor functioning
‒ Baseline associations between cardiovascular risk factors and MRI markers of cerebral structure and vascular functioning
‒ Association between circulating markers of inflammation, including cytokines and chemokines, measured at *T* = 1 and blood pressure and brain MRI parameters
‒ Changes in MRI parameters of brain structure and vascular function at 1.5-year follow-up
‒ Number of future incident clinical events. This includes all-cause mortality, death due to vascular causes, non-fatal strokes (ischemic and haemorrhagic), and TIAs.

### Cognitive assessments

At baseline, *T* = 2 and *T* = 3 patients will undergo a 60-min cognitive assessment covering six domains: processing speed, attention, executive functioning, verbal memory, working memory, and psychomotor functioning (Supplemental Table 2). All administered tests are validated and widely used. To minimize intra-individual variability, we use standardized tests with high test-retest reliability. Parallel versions will be used to take task-specific practice effects into account. Furthermore, participants will perform the Alertness subtask of the Test of Attentional Performance on a laptop during each study visit. The Alertness subtask is a sensitive test for processing speed and attention, during which participants have to press a button as quickly as possible then a target stimulus is shown.^28^

### Motor functioning

Gait speed (m/s) will be determined over a 6-m distance during each study visit to examine motor functioning. Gait and balance of participants will be assessed using the Timed Up & Go Test, measuring the time a participant needs to get up from a chair, walk 3 m, turn around, and sit back down.^29^

### Questionnaires

A structured questionnaire will be used at baseline to assess demographic data, medical history and lifestyle behavior (including smoking, alcohol consumption, and drug use). Educational level is determined using a seven-point Dutch rating scale, the Verhage scale.^30^ Medical history includes age of hypertension onset and medication use. At each follow-up visit, changes in medication use and lifestyle behavior, and incident clinical events are assessed.

### Blood sampling

Blood will be collected at *T* = 1 and *T* = 2 to determine levels of circulating inflammatory markers, such as cytokines after overnight fasting. We will collect 45 ml of blood (20 ml serum, 10 ml EDTA plasma, 9 ml citrate plasma, and 6 ml blood for DNA isolation). Samples will be stored in the Hyperintense BioBank at the Radboudumc for future analyses.

### Physical examination

We will measure height and weight and calculate the Body Mass Index (BMI). Blood pressure will be measured three times while participant is in seated position.

### Sample size calculation

Because the effects of blood pressure increase and decrease on MRI outcomes assessed here have never been examined, there is no information available about the size of these possible effects. A formal sample size calculation is therefore not feasible. Instead, we based our sample size on the number of patients referred to the Radboudumc annually that are eligible for participation in this study within the limited time period of 2.5 years.

### Statistical analysis

To analyze primary outcome measures, MRI outcomes at different timepoints will be compared. MRI outcomes are discussed in the imaging analysis section and include: WMH volume, PSMD, BBB leakage rate, BBB leakage volume, IVIM outcomes, FA, MD, network global efficiency, and clustering coefficients. All outcome measures are continuous measures and data will be log-transformed in case of non-normality. We assume a linear (or parametric) relationship between blood pressure and MRI parameters and will therefore run linear mixed models, including all time points. These will be adjusted for age, sex, education, and other conventional MRI markers of SVD. We will not correct for multiple analysis since different MRI measures probe different aspects of the brain in terms of brain structure, physiology, and function. Missing data will be described in our scientific reports.

To examine the association between changes in blood pressure and cognitive and motor functioning, individual cognitive test scores will be adjusted to *Z*-scores using available normative data,^31,32^ adjusting for age, sex, and educational levels when possible. The association between blood pressure and incident clinical events will be investigated using Cox proportional hazard analyses, adjusted for age, sex, education, and SVD MRI markers where appropriate.

## Discussion

Little is known about the pathophysiological mechanisms underlying SVD. Research into SVD pathogenesis is hampered by difficulties with visualizing the smallest cerebral vessels with conventional MRI, allowing only the detection of end-stage disease cerebral lesions. Most of the knowledge about SVD is derived from studies conducted in individuals older than 60 years in whom SVD most likely has been present for decades. To advance the understanding of key mechanisms implicated in SVD, studies conducted in young- and middle-aged adults that are able to catch the first signs of SVD, before the occurrence of widespread irreversible tissue damage, are needed. The Hyperintense study is the first serial MRI study designed to examine the effects of changes in blood pressure on cerebral microvasculature in young- and middle-aged adults with hypertension, the strongest risk factor for SVD.^1^

One of the main strengths of this study is the unique design that includes induced hypertension due to temporary withdrawal of antihypertensive medication, followed by blood pressure lowering due to medication restart as part of routine clinical practice, without the need for an intervention study. This design allows analysis of the effects of both increases as well as decreases in blood pressure. Previous studies have examined the effects of blood pressure lowering on SVD measures, but the effects of blood pressure increase have never been studied.^33^ Furthermore, we combine several advanced MRI techniques to examine early microvascular changes. We use DCE-MRI to examine BBB integrity, which is associated with clinical and imaging features of SVD.^34^ Since both microstructural integrity and cerebral perfusion are suggested to be diminished in SVD patients, we use multi-shell DWI and IVIM to examine this simultaneously.^17^ We also use rs-fMRI to examine functional connectivity since (micro)structural damage in SVD patients can lead to disturbed connectivity both between and within brain networks.^27^ These MRI techniques allow analysis of early pathological mechanisms and the order in which they occur before MRI markers of SVD become visible. Another strength of this study is the extensive amount of cognitive, motor, and biobank data that is collected in a structured and standardized way. MRI outcomes can therefore be linked to cognitive functioning, measured by tests that are widely accepted and shown to be sensitive to SVD-related brain changes. Assessment of circulating inflammatory markers will help identify ongoing inflammatory responses that may play a central role in SVD pathogenesis.^35^ We expect the external validity of this study to be high. Since the patients included in this study are highly likely to develop SVD later in life, abnormalities in vascular functioning observed here are presumably also present in other populations at risk of SVD. Findings of this study will therefore have implications for treatment optimization in patients at risk of cerebrovascular damage; reaching conventional target blood pressure may not be sufficient and additional treatment to further lower blood pressure or reduce inflammation and BBB leakage can have beneficial effects on clinical outcomes.

In conclusion, the Hyperintense study is a unique serial MRI project that has the potential to further unravel the association between early-life hypertension and SVD. Although hypertension is considered a main risk factor for developing SVD, it remains unknown how hypertension exerts an effect on brain structure and vascular function. This study will help to identify early-life pathological mechanisms of SVD caused by hypertension. Improved understanding of the pathological mechanisms driving SVD pathogenesis and progression will contribute to identification of new targets for treatment.

## Supplemental Material

sj-docx-1-eso-10.1177_23969873221100331 – Supplemental material for The Hyperintense study: Assessing the effects of induced blood pressure increase and decrease on MRI markers of cerebral small vessel disease: Study rationale and protocol Click here for additional data file. Supplemental material, sj-docx-1-eso-10.1177_23969873221100331 for The Hyperintense study: Assessing the effects of induced blood pressure increase and decrease on MRI markers of cerebral small vessel disease: Study rationale and protocol by Esther Janssen, Annemieke ter Telgte, Esmée Verburgt, Joost JA de Jong, José P Marques, Roy PC Kessels, Walter H Backes, Marnix C Maas, Frederick JA Meijer, Jaap Deinum, Niels P Riksen, Anil M Tuladhar and Frank-Erik de Leeuw in European Stroke Journal
