# Supplementary material for: The Hyperintense study: Assessing the effects of induced blood pressure increase and decrease on MRI markers of cerebral small vessel disease: Study rationale and protocol
Source: Eur Stroke J. 2022 May 12;7(3):331–8. doi: 10.1177/23969873221100331 (PMC9446329; doi:10.1177/23969873221100331)
Supplement: sj-docx-1-eso-10.1177_23969873221100331 – Supplemental material for The Hyperintense study: Assessing the effects of induced blood pressure increase and decrease on MRI markers of cerebral small vessel disease: Study rationale and protocol [file sj-docx-1-eso-10.1177_23969873221100331.docx]

Supplementary materials

**Supplementary Table 1**: *MRI acquisition parameters.*

|  | **FLAIR** | **T1w** | **B_0_ field map** | **SWI** | **DWI** | **DWI - Reversed phase encoding** |
| --- | --- | --- | --- | --- | --- | --- |
| Sequence | SPACE | 3D MP2RAGE | Dual-echo 2D SPGR | 3D SPGR | Multi-band accelerated single-shot EPI | Multi-band DW single shot EPI |
| Voxel size (mm) | 0.5×0.5×0.9 | 1.0×1.0×1.0 | 2.0×2.0×3.0 | 1.0×1.0×3.0 | 2.0×2.0×2.0 | 2.0×2.0×2.0 |
| TR (ms) | 5000 | 6000 | 531 | 27 | 4600 | 3500 |
| TE (ms) | 388 | 1.71 | 5.07, 7.53 | 20 | 80 | 66 |
| TI (ms) | 1800 | 900, 2200 |  | - | - | - |
| Flip Angle (°) | Optimized T2var refocusing | 6, 5 | 60 | 15 | 90, 180 | 90, 180 |
| Number of slices | 192 | 176 | 48 | 52 | 70 | 70 |
| Acquisition time (mm:ss) | 05:47 | 07:50 | 01:53 | 02:43 | 07:00 | 00:21 |
| Other | R = 2  Turbo factor = 278 | Also used for T1-mapping |  |  | MB = 2  R = 2  5 × b = 0 s/mm^2^, 40 × b = 1000 s/mm^2^, 40 × b = 2000 s/mm^2^) | b = 0 s/mm^2^  Phase encoding direction = P > A |

|  | **DCE** | | | **IVIM** | **rs-fMRI** |
| --- | --- | --- | --- | --- | --- |
| Sequence | Pre-bolus slow T1w SR SPGR | Fast T1w SR SPGR | Post-bolus slow T1w SR SPGR | Multi-band DW single shot EPI | Multi-band accelerated single-shot EPI |
| Voxel size (mm) | 1.0×1.0×2.0 | 1.0×1.0×3.0 | 1.0×1.0×2.0 | 2.0×2.0×2.0 | 2.0×2.0×3.0 |
| TR (ms) | 3.4 | 3.4 | 3.4 | 3500 | 1210 |
| TE (ms) | 1.56 | 1.56 | 1.56 | 66 | 30 |
| TI (ms) | 120 | 120 | 120 | - | - |
| Flip Angle (°) | 20 | 20 | 20 | 90, 180 | 60 |
| Number of slices | 60 | 10 | 60 | 70 | 48 |
| Acquisition time (mm:ss) | 01:47 | 02:00 | 24:52 | 05:32 | 08:13 |
| Other | NM = 5  R = 3 | NM = 32  R = 2  Injection of Gadobutrol | NM = 70  R = 3 | b = 5, 10, 15, 20, 30, 40, 50, 60, 100, 200, 400, 600, 800, 1000 s/mm^2^ | MB = 3  NM = 400 |

A =Anterior, DCE =Dynamic contrast-enhanced; DWI =Diffusion-weighted imaging; EPI =Echo Planar Imaging; FA =Flip Angle; FLAIR =Fluid-attenuated inversion recovery; IVIM =Intravoxel Incoherent Motion; MB =Multiband acceleration factor; MP2RAGE =Magnetization-prepared 2 rapid acquisition with gradient echo; NM =number of measurements; P = Posterior, R =parallel imaging acceleration factor; rs-fMRI =resting-state functional MRI; SPACE =Sampling perfection with application-optimized contrast using different flip-angle evolutions; SWI =Susceptibility weighted imaging; SPGR=Spoiled gradient recalled echo; SR = Saturation Recovery; TE =Echo time; TI =Inversion time; TR =Repetition time.

**Supplementary Table 2**: *Cognitive test battery administered at baseline, T=2 and T=3.*

| Cognitive domain | Cognitive test |
| --- | --- |
| Global cognitive function | Mini Mental State Examination **^1^** |
| Visuospatial episodic memory | Complex Figure Test ^2^* |
| Verbal episodic memory | Rey Auditory Verbal Learning Test ^3^* |
| Information processing speed | Trail Making Test Part A ^4^ |
| Executive function (mental flexibility) | Trail making Test Part B ^4^ |
| Executive function (semantic fluency) | Verbal fluency: animal and profession naming |
| Information processing speed | Symbol Digit Modalities Test (written version) ^5^ |
| Language (naming) | Boston Naming test (CERAD version) ^6^ |
| Working memory | Digit Span Test (forward & backward) ^7^ |

* parallel versions of test used at each follow-up to avoid learning effects

Supplementary references

1. Cockrell JR and Folstein MF. Mini-Mental State Examination (Mmse). *Psychopharmacol Bull* 1988; 24: 689-692.

2. Shin MS, Park SY, Park SR, et al. Clinical and empirical applications of the Rey-Osterrieth Complex Figure Test. *Nat Protoc* 2006; 1: 892-899.

3. Van der Elst W, Van Boxtel MPJ, Van Breukelen GJP, et al. Rey's verbal learning test: Normative data for 1855 healthy participants aged 24-81 years and the influence of age, sex, education, and mode of presentation. *J Int Neuropsych Soc* 2005; 11: 290-302.

4. Bowie CR and Harvey PD. Administration and interpretation of the trail making test. *Nat Protoc* 2006; 1: 2277-2281.

5. Smith A. *Symbol Digit Modalities Test; Handleiding.* 2010. Amsterdam.

6. Strauss E, Sherman EM and Spreen O. *A compendium of neuropsychological tests: Administration, norms, and commentary*. American Chemical Society, 2006.

7. Wechsler D. WAIS-IV-NL: Wechsler adult intelligence scale--Nederlandstalige bewerking: Technische handleiding en Afname en scoringshandleiding. *BV APAaI, editor* 2012.
